# CFD-Derived Regression Model to Predict Surface Velocity for a Continuous-Casting Round Billet Mold

**DOI:** 10.3390/ma19153309

**Published:** 2026-08-04

**Authors:** Guangchao Guo, Jiangshan Zhang, Mengjing Zhao, Shufeng Yang, Xiaotan Zuo, Qing Liu

**Affiliations:** 1 State Key Laboratory of Advanced Metallurgy, University of Science and Technology Beijing, Beijing 100083, China; ggc15890352855@126.com (G.G.); yangshufeng@ustb.edu.cn (S.Y.); 2 Metallurgical and Building Materials Business Department, China International Engineering Consulting Corporation, Beijing 100048, China; ustbzhaomengjing@163.com; 3 Wuhu Xinxing Ductile Iron Pipes Co., Ltd., Wuhu 241002, China; zuoxiaotan345@163.com

**Keywords:** regression model, round billet mold, surface velocity, high-velocity range, real-time prediction

## Abstract

**Highlights:**

- A 3D electromagnetic–fluid dynamic model was established to simulate mold surface flow in continuous casting.
- Predictive formulas for maximum surface velocity and high-velocity range were derived under varying process parameters.
- A CFD-derived regression model predicts key surface-flow characteristics within milliseconds with satisfying accuracy.

**Abstract:**

In the continuous casting process, the flow velocity of molten steel at the surface of the mold directly influences the melting of the flux, slag entrainment behavior, and the uniformity of heat transfer. Fast and accurate prediction of the magnitude and distribution of surface-flow velocity is crucial for defect suppression and ensuring continuous casting quality. In this study, a three-dimensional electromagnetic–fluid dynamic coupled model was established and validated to simulate the molten steel flow behavior under various casting speeds, Electromagnetic Stirring (EMS) currents, EMS frequencies, and Submerged Entry Nozzle (SEN) immersion depth. Subsequently, the maximum surface velocity in the mold was fitted to derive a predictive formula under different conditions; the median range and width of the surface high-velocity range were predicted in a similar way. As a result, a regression model derived from Computational Fluid Dynamics (CFD) was built to predict the maximum mold surface velocity and the high-velocity range within milliseconds. The reliability of the regression model was verified by newly generated simulation results, with the relative error between the predicted and simulated maximum surface velocities kept within 2%. It was shown that the maximum surface velocity increases with casting speed and EMS current but decreases with EMS frequency. The median range of the high-velocity range decreases with increasing casting speed and current. The immersion depth of SEN had a minimal impact on the maximum surface velocity in the mold and the high-velocity range. This regression model is beneficial for rapid mold design and for predicting potential slag entrainment, breakout risks, and billet defects, for the purpose of intelligent continuous casting.

## 1. Introduction

In the modern steelmaking industry, continuous casting works as a critical process in steel production and significantly influences product quality and efficiency. As a pivotal component of the continuous casting process, the mold involves complex phenomena encompassing fluid flow, heat transfer, and physicochemical reactions. Among these, the flow behavior of molten steel plays a decisive role, directly affecting mold flux melting, slag entrainment, and heat transfer uniformity [1,2,3,4,5,6,7,8]. Firstly, regarding mold flux melting, an appropriate surface-flow velocity facilitates the rapid transformation of solid powder flux into a liquid slag layer, ensuring the continuous formation and replenishment of the liquid slag. This is essential for maintaining effective lubrication and thermal insulation properties [9]. Secondly, the stability of the flow field directly determines the extent of interfacial fluctuations. Excessive turbulence or improper vortices can entrain liquid mold flux into the bulk steel, leading to potential inclusion defects [10]. Furthermore, the flow state of the molten steel governs the heat exchange efficiency between the high-temperature steel and the mold copper walls. A uniform flow-field distribution prevents local overheating or uneven cooling, thereby ensuring the uniform growth of the solidified shell of the cast strand [9,11,12].

With the rapid advancement of computer technology, numerical simulation has found increasingly widespread application in the field of metallurgy. Surface-flow behavior has been frequently investigated in continuous casting molds through computational fluid dynamics (CFD) simulation. However, the traditional CFD workflow encompasses the entire process of pre-processing, solving, and post-processing, which can take days to weeks for one case. For instance, Cui et al. [13] investigated flow behavior within the mold via numerical simulation, where completing a single transient multi-physics simulation required 30–40 h. Similarly, during the optimization design of submerged entry nozzles (SENs), Qin et al. [14] reported that the CFD calculation time for each operating condition exceeded 48 h. Such prohibitive time costs render traditional CFD methods inadequate for meeting the demands of rapid process adjustment, real-time optimization control, or multi-parameter batch optimization in actual production. This is particularly true in scenarios requiring iterative design, where computational efficiency has become the primary bottleneck restricting engineering applications. To overcome this bottleneck, constructing simplified predictive models—specifically regression models—from CFD simulation databases offers a practical solution. By establishing mapping relationships between input parameters (such as casting speed, SEN structural parameters, electromagnetic stirring current, and frequency) and output responses (such as surface velocity and jet impact depth), these models can reduce prediction time from hours to seconds or even milliseconds, while maintaining acceptable accuracy [15,16]. For example, Zhou et al. [17,18] fitted formulas to predict the maximum surface velocity, the coordinate value of the vortex position, and the critical relationship for the transition of the flow pattern under varying parameters. These predictive formulas were employed to optimize the mold flow field online, successfully controlling the slag entrainment defect rate in ultra-low carbon steel to below 3%. Huang et al. [19] developed a data-driven prediction framework to achieve rapid prediction of multi-physics fields within the mold; the average computational time was drastically reduced from 24 h to just 2 s, with prediction errors for flow and temperature fields remaining within 10%. Liu et al. [20] employed a Weighted Extreme Learning Machine (WELM) to address data imbalance issues in cast slab quality prediction. Their approach maintained robust predictive performance and generalization capability even when training on massive datasets, effectively meeting the requirements for slab quality prediction. Furthermore, to meet the need for real-time assessment of melt pool velocity distribution, Wang et al. [21] substituted traditional numerical models with deep learning algorithms. Their results showed a prediction accuracy of 93.12% with a computation time of merely 0.12 s. While these data-driven approaches have demonstrated impressive computational efficiency, they typically rely on extensive training datasets and complex model architectures. This study employs an alternative approach. Physics-informed parametric correlations are constructed directly from a systematically generated CFD database, thereby linking several key operating parameters—such as casting speed, EMS current and frequency, and SEN immersion depth—to scalar surface-flow indicators. This approach offers a more transparent and computationally lightweight solution. Meanwhile, to the best of our knowledge, existing research in this area has predominantly focused on slab continuous casting, and studies dedicated to regression models for predicting surface-flow velocity in round billet molds remain undisclosed, particularly regarding the characterization of surface velocity distribution.

In light of the literature, this study aims to develop a CFD regression model specifically for predicting surface-flow velocity in round billet molds. The research methodology proceeds as follows: first, a comprehensive flow-field dataset for a round billet mold is established through systematic CFD numerical simulations, covering a wide range of operating conditions, including casting speed, electromagnetic stirring (EMS) current and frequency, and SEN immersion depth. Subsequently, by fitting the data within this database, predictive formulas are derived for the maximum surface velocity and high-velocity ranges under varying parameters. Finally, the prediction accuracy of the proposed regression model is validated against unknown operating conditions. This study sets out to provide an efficient and reliable method for rapid surface-flow velocity prediction in round billet continuous casting, offering technical support for real-time process parameter adjustment and optimization decision-making during production.

## 2. Mathematical Model

The fundamental controlling equations of fluid flow in the mold include continuity, momentum, and *k–ε* equations. The equations governing electromagnetic fields are Maxwell’s equations, constitutive equations, and Ohm’s law.

### 2.1. Fluid Flow Equations

Continuity equation:

(1) $$\frac{\partial \left(\rho u_{j}\right)}{\partial x_{j}}+\frac{\partial \rho }{\partial t}=0$$

Momentum equation:

(2) $$\rho \frac{\partial u_{i}}{\partial t}+\rho \frac{\partial u_{i}u_{j}}{\partial x_{j}}=-\frac{\partial p}{\partial x_{j}}+\frac{\partial }{\partial x_{j}}\left[\mu_{eff}\left(\frac{\partial u_{i}}{\partial x_{j}}+\frac{\partial u_{j}}{\partial x_{i}}\right)\right]+\rho g_{i}+F_{E}$$

where $u_{i}$ and $u_{j}$ represent the velocity components in the $x_{i}$ and $x_{j}$ directions, m s^−1^; ρ is the fluid density, kg m^−3^; $\mu_{eff}$ is the effective viscosity, kg m^−1^ s^−1^; $F_{E}$ is the source term for the electromagnetic force, N m^−3^; p is the pressure, Pa; and $g_{i}$ is the gravitational acceleration, m s^−2^.

The standard *k–ε* model is employed to describe turbulence:

(3) $$\frac{\partial \left(\rho k\right)}{\partial t}+\frac{\partial \left(\rho u_{i}k\right)}{\partial x_{i}}=\frac{\partial }{\partial x_{j}}\left|\left(\mu +\frac{\mu_{t}}{\sigma_{k}}\right)\frac{\partial k}{\partial x_{j}}\right|+G_{k}-\rho \varepsilon$$

(4) $$\frac{\partial \left(\rho \varepsilon \right)}{\partial t}+\rho \frac{\partial \left(\rho \varepsilon u_{i}\right)}{\partial x_{i}}=\frac{\partial }{\partial x_{j}}\left|\left(\mu +\frac{\mu_{t}}{\sigma_{\varepsilon }}\right)\frac{\partial \varepsilon }{\partial x_{j}}\right|+C_{1\varepsilon }\frac{\varepsilon }{k}G_{k}-C_{2\varepsilon }\frac{\varepsilon^{2}}{k}\rho$$

where $G_{k}$ and $\mu_{t}$ are expressed as

(5) $$G_{k}=\mu_{t}\frac{\partial u_{j}}{\partial x_{i}}\left(\frac{\partial u_{i}}{\partial x_{j}}+\frac{\partial u_{j}}{\partial x_{i}}\right)$$

(6) $$\mu_{t}=\rho C_{u}\frac{k^{2}}{\varepsilon }$$

where, $C_{1\varepsilon }$, $C_{2\varepsilon }$, $C_{u}$, $\sigma_{k}$ and $\sigma_{\varepsilon }$ are 1.44, 1.92, 0.09, 1.0, and 1.3, respectively [22].

### 2.2. Electromagnetic Governing Equations

The Lorentz force is expressed as follows:

(7) $$F=\vec{J}\times \vec{B}$$

Constitutive equation:

(8) $$\vec{B}=\mu \vec{H}$$

Ohm’s law:

(9) $$\vec{J}=\sigma (\vec{E}+\vec{v}\times \vec{B})$$

Ampere’s law:

(10) $$\nabla \times \vec{H}=\vec{J}$$

Since the EMS operates at low frequencies (1–4 Hz), the ratio $\omega \varepsilon_{0}/\sigma$ ≪ 1, making the displacement current term in Ampere’s law negligible.

Faraday’s law:

(11) $$\nabla \times \vec{E}=-\frac{\partial \vec{B}}{\partial t}$$

Gauss’s law for magnetism:

(12) $$\nabla \cdot \vec{B}=0$$

where $\vec{H}$ is the magnetic field intensities, A m^−1^; F is the Lorentz force density, N m^−3^; $\vec{E}$ is the electric field intensities, V m^−1^; $\vec{J}$ is the current density, A m^−2^; $\vec{B}$ is the magnetic flux, T; $\vec{v}$ is the velocity vector of the molten steel, m s^−1^; σ is the electrical conductivity, S m^−1^; μ is the magnetic permeability, H m^−1^; ω is the angular frequency, rad s^−1^; and $\varepsilon_{0}$ is the vacuum permittivity, F m^−1^.

### 2.3. Geometric Structure and Physical Properties

**Figure 1:**
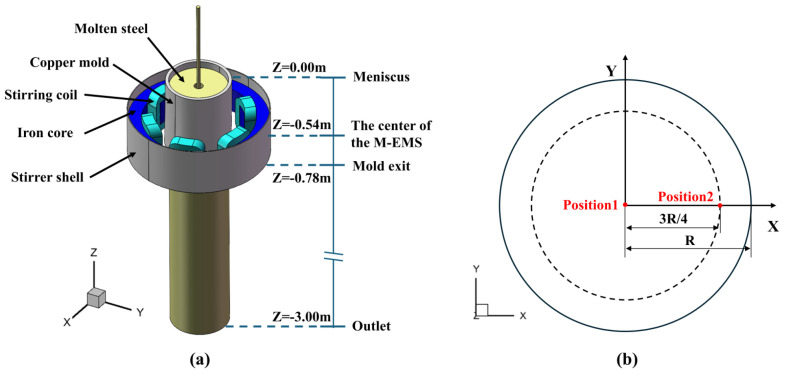
Geometric model of the electromagnetic stirrer and the mold: (**a**) 3D model; (**b**) XY-plane view of the model.

A Φ 600 mm round billet mold was studied, which is equipped with a single-port SEN. The geometric model of the mold and the electromagnetic stirrer are presented in Figure 1, and the corresponding geometric dimensions and physical properties are listed in Table 1.

### 2.4. Assumptions and Boundary Conditions

The accuracy of the simulation depends critically on reasonable assumptions and proper boundary conditions. The flow field in the mold is modeled in ANSYS Fluent 2023 R1, and the magnetic field is solved in ANSYS Maxwell 2023 R1. The resulting magnetic field data are then coupled to the flow solver via Fluent MHD module [14].

To simplify the computational process, the following reasonable assumptions were made:

(1) The molten steel within the mold is considered a homogeneous incompressible fluid, with constant physical properties.
(2) The effects of protective slag and mold oscillation are neglected. Isothermal conditions are assumed, and no phase change or mushy zone are considered.
(3) The curvature of the caster centerline is neglected; the mold is treated as a straight vertical section.
(4) No chemical reactions are assumed to occur.
(5) Temperature effects are disregarded.

The boundary conditions for the mathematical model are shown in Table 2. The inlet boundary condition was set as a velocity inlet, with boundary values determined by mass conservation, taking into account both the casting speed and the cross-sectional dimensions of the mold. The outlet of the computational domain was defined as a pressure outlet boundary. The upper surface of the computational domain was designated as a slip wall boundary, where the shear stress component is zero [23].

A pressure-based solver was employed utilizing the PISO algorithm for pressure-velocity coupling. The Reynolds-averaged Navier–Stokes (RANS) method was selected to simulate the flow inside the mold, primarily due to its proven accuracy in engineering applications coupled with its relatively low computational resource demands. Specifically, the standard *k–ε* turbulence model, a variant of the RANS method, was adopted for the mold simulation in conjunction with standard wall functions. The computed wall y+ values range from 0.096 to 267.83, with an area-weighted average of 80.50. The majority of the wall region exhibits y+ values within the ideal range of 30–300, satisfying the applicability conditions of standard wall functions. A small number of regions with y+ < 30, mainly located near the mold wall outlet, have a limited impact on the overall velocity prediction. Spatial discretization was performed using the second-order upwind scheme for momentum and the first-order upwind scheme for turbulence equations and magnetic field components. A second-order scheme was used for pressure. First-order implicit formulation was employed for temporal discretization. The MHD coupling was implemented via the magnetic induction method, accounting for two-way interaction between the flow and magnetic fields. The computational mesh consisted of approximately 503,550 hexahedral cells with a minimum orthogonal quality of 0.76. The convergence criterion for the numerical model was that the variable residuals were less than 1 × 10^−4^. To maintain the Courant numbers of the mesh and interfaces within a reasonable range (less than 2), a time step of 0.001 s was chosen for solving the transient turbulent flow. The convergence of the flow field was monitored by tracking the velocity near the meniscus surface, which stabilized within the 110 s simulation time. Under EMS conditions, the flow exhibited quasi-periodic behavior consistent with the magnetic field. The simulations were executed in parallel using a 56-core Intel Xeon Gold 6258R CPU (2.7 GHz). On average, a multi-physics transient simulation case took 48-60 h to complete, with the required time potentially varying based on different conditions.

## 3. Model Validation

**Figure 2:**
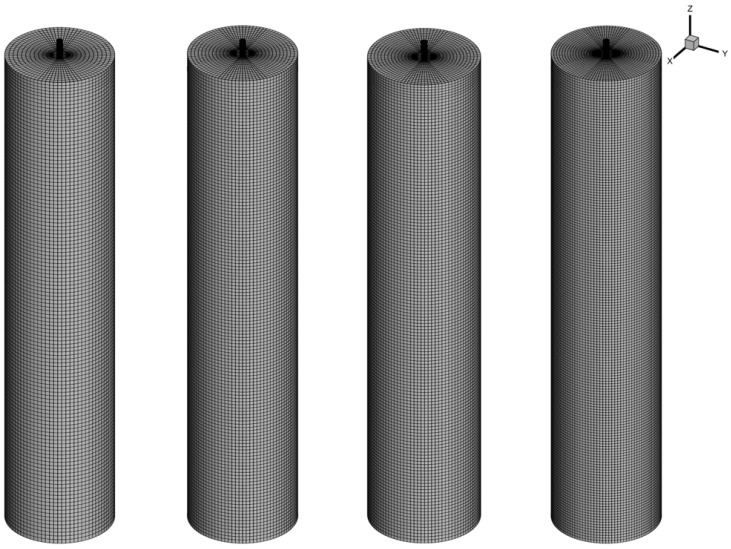
Mold mesh with different numbers of grid cells.

This study employed ANSYS ICEM CFD to generate structured grids, with local refinement applied to regions with high velocities and large velocity gradients, including the SEN inlet, boundaries, and the molten steel, as shown in Figure 2. To ensure simulation accuracy, a mesh independence study was conducted. As this research primarily focuses on the flow behavior at the mold surface, the maximum velocity at the mold surface was selected as the evaluation object. The basic parameters were set as follows: a magnetic field strength of 0 mT, a casting speed of 0.26 m/min, and a SEN immersion depth of 100 mm. As shown in Table 3, the simulation error statistics for the maximum mold surface velocity with different grid numbers are presented. When the grid number increased from 503,550 to 756,204, the results indicated that the relative error of the maximum velocity decreased from 9.15% to 4.52%, which is less than 5% [13]. Considering the balance between computational cost and simulation accuracy, this study adopted a grid system M_3_ with 503,550 hexahedral cells.

**Figure 3:**
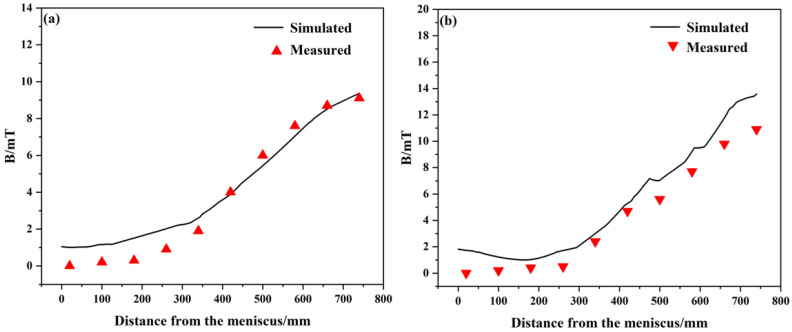
Comparison between simulated and measured magnetic flux density: (**a**) position1; (**b**) position2.

This study simulated the magnetic field inside the mold using ANSYS Maxwell. Concurrently, a Gauss meter was used to measure the magnetic field at two designated positions within the mold: the center (Position1) and 3/4R (Position2), as depicted in Figure 1b. A comparison was then made between the simulated and experimentally measured magnetic flux densities at different positions, with the EMS operating at a current of 150 A and a frequency of 2 Hz. As shown in Figure 3, the comparison between the simulated and measured magnetic flux density indicates a good agreement. However, certain deviations were observed, which are attributed to unavoidable human operational errors during on-site measurements and the inherent precision limitations of the Gauss meter [14].

It should be noted that the CFD model has been validated only for the magnetic field distribution. Direct experimental validation of the surface velocity was not performed in this study, and the velocity predictions reported herein should therefore be interpreted as numerical results consistent with the validated electromagnetic model.

## 4. Results and Discussion

### 4.1. Velocity Distribution of Molten Steel in the Mold

**Figure 4:**
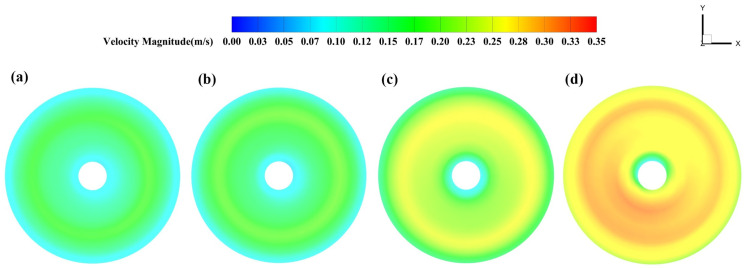
Free-surface velocity distribution of the mold at different casting speeds: (**a**) 0.26 m/min; (**b**) 0.3 m/min; (**c**) 0.5 m/min; (**d**) 1.0 m/min.

**Figure 5:**
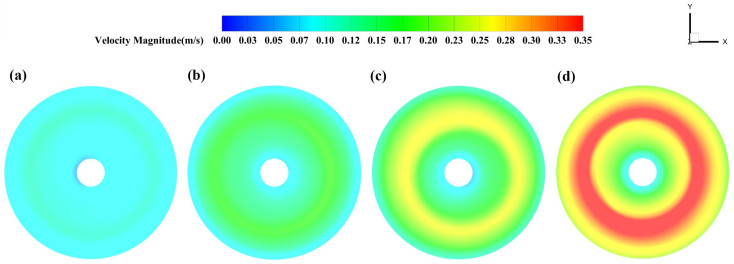
Free-surface velocity distribution of the mold at different EMS current intensities: (**a**) 100 A; (**b**) 150 A; (**c**) 200 A; (**d**) 300 A.

**Figure 6:**
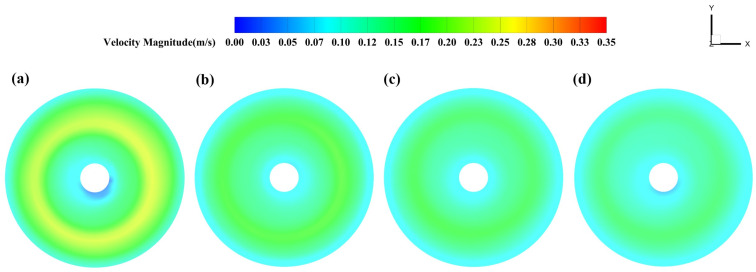
Free-surface velocity distribution of the mold at different EMS frequencies: (**a**) 1 Hz; (**b**) 2 Hz; (**c**) 3 Hz; (**d**) 4 Hz.

Figure 4, Figure 5 and Figure 6 depict the velocity distribution of the mold free surface under varying casting speeds, EMS currents, and EMS frequencies, respectively. It is evident that these parameters exert a significant influence on the surface-flow velocity within the mold. As illustrated in Figure 4, with an electromagnetic stirring current of 150 A, a frequency of 2 Hz, and a submerged nozzle depth of 100 mm, an increase in casting speed progressively elevates the free-surface flow velocity in the mold. Moreover, at higher casting speeds, a substantial enhancement in velocity is observed across the entire free surface. At a casting speed of 0.26 m/min, the maximum surface velocity reaches 0.184 m/s, which increases to 0.316 m/s when the casting speed is raised to 1.0 m/min. In Figure 5, with a casting speed of 0.26 m/min, an electromagnetic stirring frequency of 2 Hz, and a submerged nozzle depth of 100 mm, increasing the current gradually enhances the free-surface flow velocity of the mold. Unlike the effect of casting speed, as the current increases, the flow velocity near the edges of the mold surface increases significantly, while the velocity near the central submerged nozzle shows minimal change. At a current of 100 A, the maximum surface velocity is 0.133 m/s, increasing to 0.378 m/s when the current is raised to 300 A. Figure 6 shows that with a casting speed of 0.26 m/min, an electromagnetic stirring current of 150 A, and a submerged nozzle depth of 100 mm, an increase in frequency leads to a gradual decrease in the free-surface flow velocity of the mold. At a frequency of 1 Hz, the maximum surface velocity is 0.227 m/s, which decreases to 0.161 m/s when the frequency is increased to 4 Hz. Overall, a higher casting speed results in a greater mold surface velocity. Conversely, higher currents and lower frequencies intensify the electromagnetic stirring effect, leading to an increased mold surface-flow velocity.

**Figure 7:**
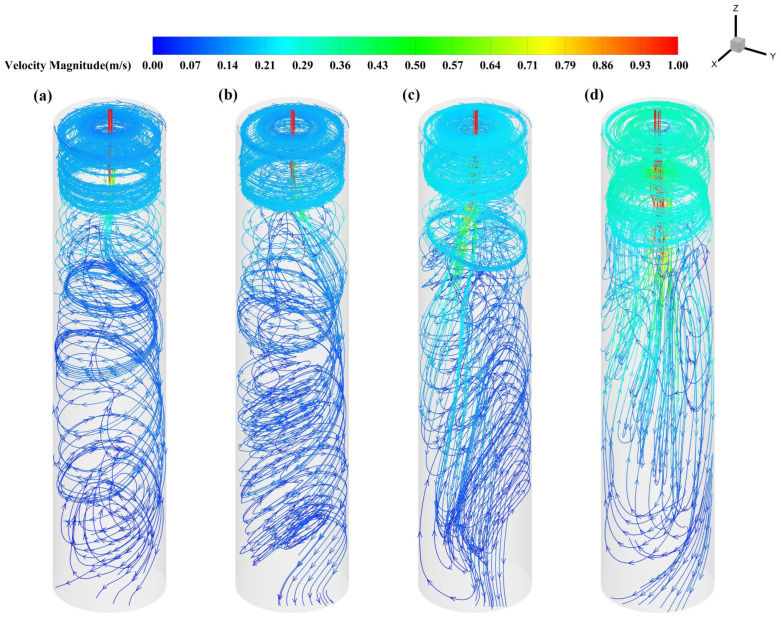
Velocity distribution in the mold at different casting speeds: (**a**) 0.26 m/min; (**b**) 0.3 m/min; (**c**) 0.5 m/min; (**d**) 1.0 m/min. The arrows indicate the flow direction.

**Figure 8:**
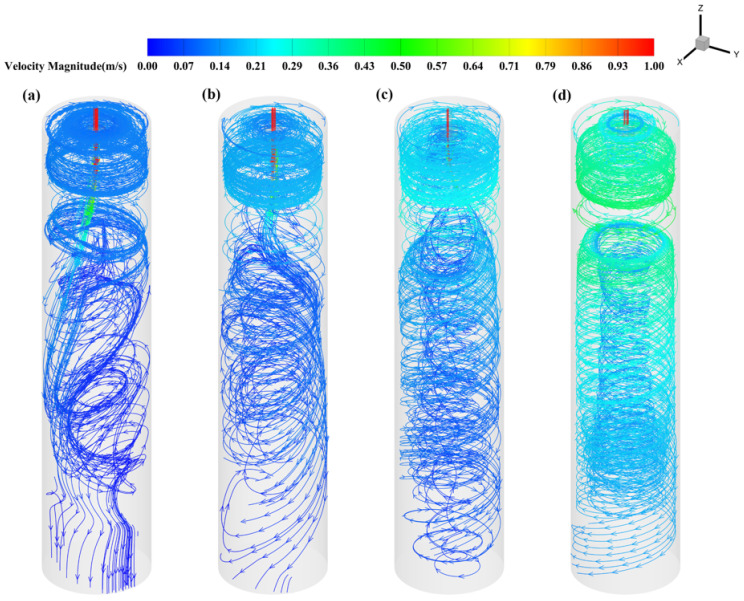
Velocity distribution in the mold at different EMS current intensities: (**a**) 100 A; (**b**) 150 A; (**c**) 200 A; (**d**) 300 A. The arrows indicate the flow direction.

**Figure 9:**
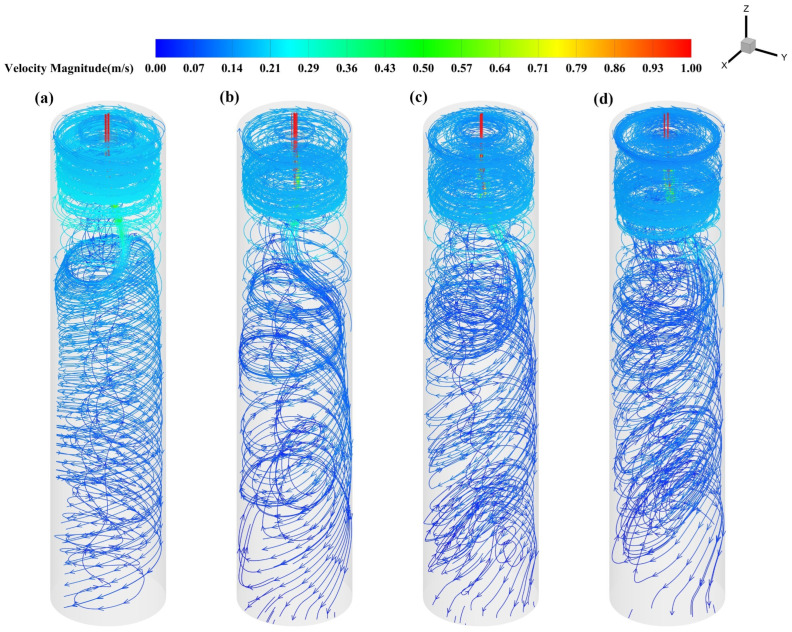
Velocity distribution in the mold at different EMS frequencies: (**a**) 1 Hz; (**b**) 2 Hz; (**c**) 3 Hz; (**d**) 4 Hz. The arrows indicate the flow direction.

Figure 7, Figure 8 and Figure 9 illustrate the velocity distributions within the mold under varying casting speeds, EMS currents, and EMS frequencies, respectively, with other parameters held constant as in the surface velocity distribution analysis. It can be observed that the molten steel exhibits a swirling flow pattern within the mold, and the flow velocity is relatively higher in the electromagnetic stirring zone. As depicted in Figure 7, an increase in casting speed leads to a gradual rise in the internal flow velocity and a deepening of the impingement depth of molten steel. The flow-field distribution becomes more chaotic, which might be attributed to the accelerated downward movement of molten steel at higher casting speeds, causing the rotational flow or horizontal circulation induced by electromagnetic stirring to expand over a larger area [24]. Figure 8 shows that as the current increases, the flow velocity within the mold gradually rises, the impingement depth of molten steel lessens, and the flow-field distribution becomes more uniform. Concurrently, it is evident that the swirling motion of the molten steel becomes more pronounced with increasing current, displaying a phenomenon where the flow velocity is higher in the outer edges and lower in the central regions. This could be due to the magnetic field strength decaying with distance. Such an externally strong and internally weak flow pattern is beneficial for promoting inclusion flotation, scouring the initial solidified shell, and improving temperature uniformity. However, excessively high velocities in the wall regions might also lead to slag entrainment [25]. Figure 9 indicates that as the frequency increases, the flow velocity within the mold gradually decreases, and the flow-field distribution becomes more chaotic. The primary reason for this might be that an increased frequency reduces the skin depth of electromagnetic stirring, consequently diminishing the overall stirring intensity and uniformity [26].

### 4.2. Prediction of Maximum Surface Velocity

**Figure 10:**
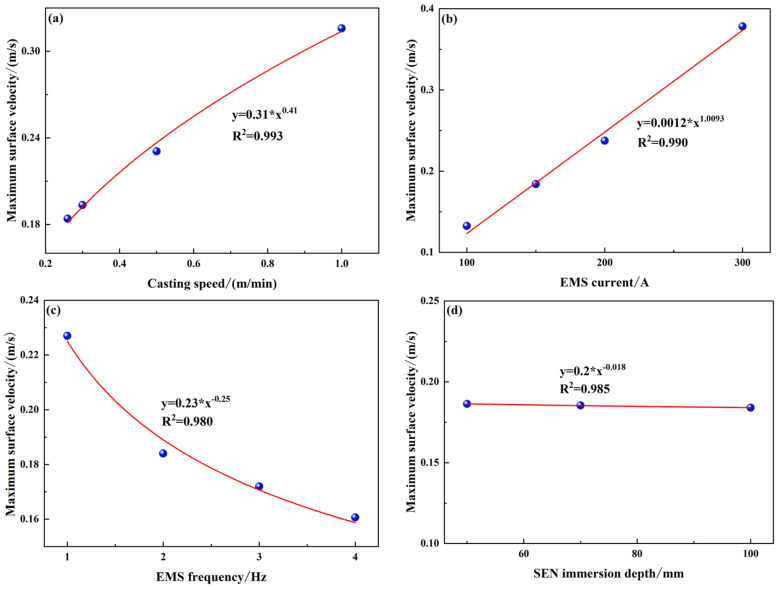
Fitting of maximum surface velocity in the mold under different casting parameters: (**a**) casting speed; (**b**) EMS current; (**c**) EMS frequency; (**d**) SEN immersion depth.

Figure 10 displays the fitted results for the maximum surface velocity of the mold under various casting parameters. The maximum mold surface velocity increases with increasing casting speed and EMS current, while it decreases with increasing EMS frequency. The effect of SEN immersion depth on the maximum surface velocity is minimal. Based on the fitting results for casting speed, current, frequency, and SEN immersion depth, a regression equation for predicting the maximum velocity at the mold free surface is obtained as follows:

(13) $$v_{max}=C\times v^{0.407}\times I^{1.009}\times f^{-0.252}\times H^{-0.0184}$$

where $v_{max}$ is the maximum surface velocity of the mold, m/s; C is a coefficient; v is the casting speed, m/min; I is the EMS current, A; f Is the EMS frequency, Hz; and H is the SEN immersion depth, mm.

**Figure 11:**
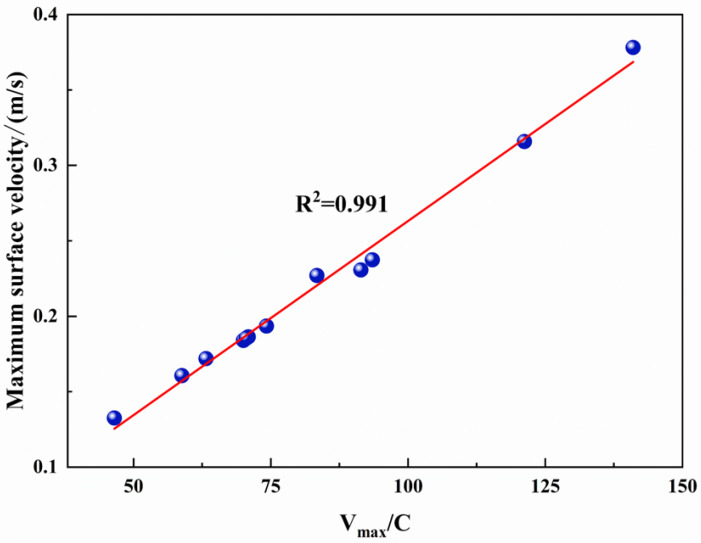
Coefficient for the regression formula of maximum mold surface velocity.

The values for different casting speeds, currents, frequencies, and SEN immersion depth were substituted into Equation (14) to compute the $v_{max}/C$ ratio. Subsequently, the maximum surface velocity was fitted to ascertain the coefficient C. It is noted that all variables in the formula must be used with the specified engineering units, and only under this unit system can the numerical value of C be directly applied. The fitting results are shown in Figure 11. As a result, the regression equation for calculating the maximum surface velocity of the mold is obtained as follows:

(14) $$v_{max}=0.00257\times v^{0.407}\times I^{1.009}\times f^{-0.252}\times H^{-0.0184}$$

To enhance the applicability of the predictive method, the flow velocity prediction equations for round billet molds can be normalized. It should be noted that the normalization based on the mold radius *R* in Equation (15) was performed solely using the simulation data of the Φ 600 mm mold. The applicability of this normalized form to other sizes of round billet molds remains to be verified in future studies. Consequently, the following regression equations for the maximum surface velocity of the mold are obtained:

(15) $$v_{max}=\left(0.00857\;{\times \;v}^{0.407}\times I^{1.009}\times f^{-0.252}\times H^{-0.0184}\right)R$$

where *R* is the radius of the round billet mold, m.

### 4.3. Prediction of High-Velocity Ranges near the Free Surface

**Figure 12:**
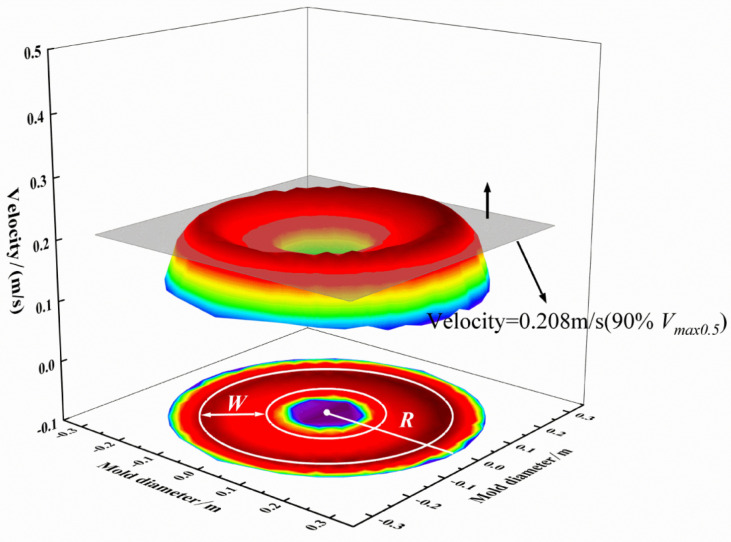
Three-dimensional schematic of surface velocity in the mold at a casting speed of 0.5 m/min.

In practical production, excessive surface velocity or unstable flow patterns within the mold significantly elevate the risk of slag entrainment. Even when the overall surface steel flow velocity is relatively low, substantial velocity gradients can disrupt the continuity of the slag layer and lead to entrainment [24,27]. Therefore, precisely identifying regions of high velocity is crucial for targeted optimization of process parameters, thereby mitigating such defects. Analysis of the mold free-surface flow field under various parameters revealed a high-velocity annular region, as typically shown in Figure 12 for a casting speed of 0.5 m/min, an EMS current of 150 A, an EMS frequency of 2 Hz, and an SEN immersion depth of 100 mm. This high-velocity region, denoted as *W*, shows an approximately annular shape with relatively high surface-flow velocities throughout. If the maximum surface velocity under this condition is *V_max_*_0.5_, the critical velocity within the high-velocity range approximates 90% of the maximum surface velocity. We would like to point out that the ‘high-velocity region’ serves merely as a descriptive feature of the velocity distribution. The actual risk of slag entrainment must be evaluated in conjunction with specific critical velocity thresholds. Under certain operating conditions with relatively low *V_max_*, although a high-velocity region may still exist, the practical risk of slag entrainment could be relatively low. We have also compiled the high-velocity ranges across different casting parameters, as presented in Table 4, where *R* denotes the radius of the round billet mold, which is 0.3 m in this study.

**Figure 13:**
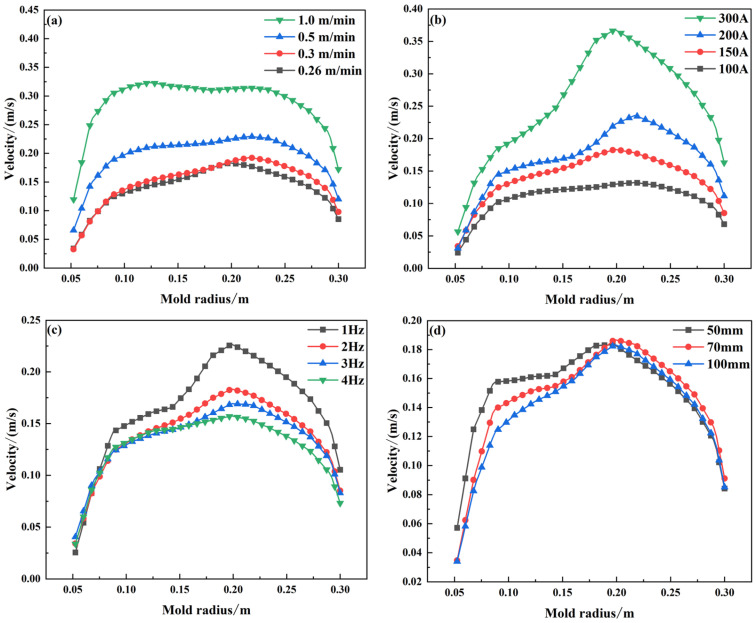
Velocity distribution at the free surface of the mold under different casting parameters: (**a**) casting speed; (**b**) EMS current; (**c**) EMS frequency; (**d**) SEN immersion depth.

Figure 13 presents the free surface flow velocity distribution along a typical radius under various casting parameters. It can be observed that across the radial range from the mold center to the wall, the surface-flow velocity generally increases with increasing casting speed, increasing current, and decreasing frequency. However, the degree of influence of these casting parameters on the flow velocity distribution exhibits some variation. As illustrated in Figure 13a, an increase in casting speed leads to a larger region at the free surface experiencing higher flow velocities. Specifically, at a casting speed of 1.0 m/min, the high-velocity range width extends beyond 50% of the mold radius, with a notable acceleration of flow velocity in proximity to the SEN. Figure 13b,c demonstrate that as the current increases and the frequency decreases, the mold surface-flow velocity progressively rises, and the flow velocity peaks become more pronounced. Furthermore, the centers of these high-velocity ranges tend to shift towards the SEN. Regarding the SEN immersion depth, as shown in Figure 13d, variations in immersion depth primarily affect the flow velocity in the region close to the SEN. A decrease in SEN immersion depth results in a gradual increase in flow velocity near the SEN, while exerting a minimal impact on the high-velocity range at the free surface of the mold.

**Figure 14:**
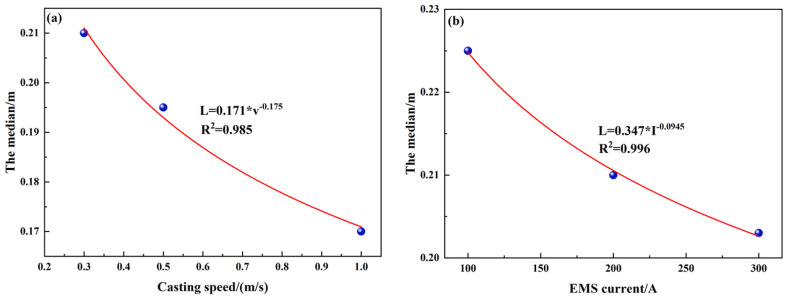
Fitting of the median of the high-velocity range at the mold free surface under different casting parameters: (**a**) casting speed; (**b**) EMS current.

**Figure 15:**
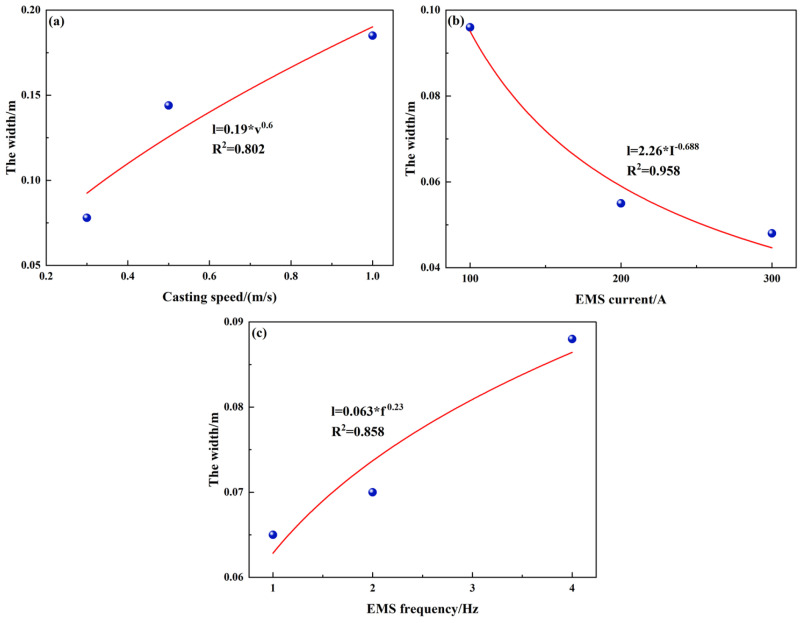
Fitting of the width of the high-velocity range at the mold free surface under different casting parameters: (**a**) casting speed; (**b**) EMS current; (**c**) EMS frequency.

Figure 14 and Figure 15 present the fitting results for the median value and width of the high-velocity range at the free surface under different casting parameters. To ensure a satisfactory fitting result, data points with significant deviations were excluded, and a subset of data was selected for fitting the high-velocity range predictions. Within the parameter range of this study, the influence of frequency on the median range was not significant and a constant value of 0.204 m was used. Additionally, the SEN immersion depth had a minor effect on the high-velocity range. As observed in Figure 14, an increase in casting speed and current led to a gradual decrease in the median range of the high-velocity range, indicating a trend towards proximity to the SEN. Figure 15 reveals that the width of the high-velocity range increases with casting speed but decreases with increasing current. This reduction in width with increasing current is primarily attributed to a significant expansion of the peak velocity region at the free surface, resulting in steeper velocity peaks and a narrower high-velocity range. Conversely, an increase in frequency leads to a gradual widening of the high-velocity range, as the surface velocity decreases and the velocity peaks become more moderate. Based on the fitting results for casting speed, current, and frequency, regression equations for the median and width of the high-velocity range at the mold free surface are obtained as follows:

(16) $$L=C_{1}\;{\times \;v}^{-0.175}\times I^{-0.0945}$$

(17) $$l=C_{2}{\;\times \;v}^{0.6}\times I^{-0.688}\times f^{0.23}$$

where L is the median of the high-velocity range, m; l is the width of the high-velocity range, m; $C_{1}$ and $C_{2}$ are coefficients; v is the casting speed, m/min; I is the EMS current, A; and f is the EMS frequency, Hz.

**Figure 16:**
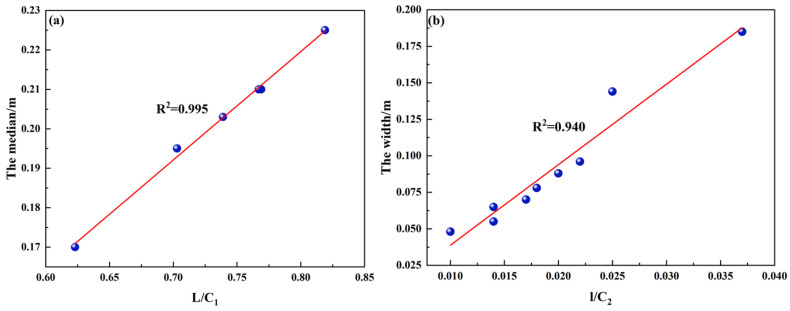
Determination of coefficients for the regression equations of the median and width of the high-velocity range at the mold free surface: (**a**) the median; (**b**) the width.

Substituting different casting speeds, currents, and frequencies back into Equations (16) and (17), the ratios $L/C_{1}$ and $l/C_{2}$ were calculated. Similarly, these ratios were then fitted against the median and width of the high-velocity range, respectively, to determine the coefficients $C_{1}$ and $C_{2}$. The fitting results are shown in Figure 16. Thus, the regression equations for calculating the median and width of the high-velocity range at the mold free surface are obtained as follows:

(18) $$L=0.275{\;\times \;v}^{-0.175}\times I^{-0.0945}$$

(19) $$l=5.51{\;\times \;v}^{0.6}\times I^{-0.688}\times f^{0.23}$$

Similarly, the median L and width l of the high-velocity range are also normalized by the mold radius R, with the same limitation regarding cross-size applicability. The following regression equations for the median and width of the high-velocity range at the mold free surface are obtained:

(20) $$L=\left(0.917{\;\times \;v}^{-0.175}\times I^{-0.0945}\right)R$$

(21) $$l=\left(18.4{\;\times \;v}^{0.6}\times I^{-0.688}\times f^{0.23}\right)R$$

Accordingly, the mathematical expression for the high-velocity range at the mold free surface is given as follows:

(22) $$E_{W}=0.275{\;\times \;v}^{-0.175}\times I^{-0.0945}\pm 2.755{\;\times \;v}^{0.6}\times I^{-0.688}\times f^{0.23}$$

(23) $$E_{W}=\left(0.917{\;\times \;v}^{-0.175}\times I^{-0.0945}\pm 9.18{\;\times \;v}^{0.6}\times I^{-0.688}\times f^{0.23}\right)R$$

where $E_{W}$ is the high-velocity range, m.

### 4.4. Validation of the Regression Model

**Figure 17:**
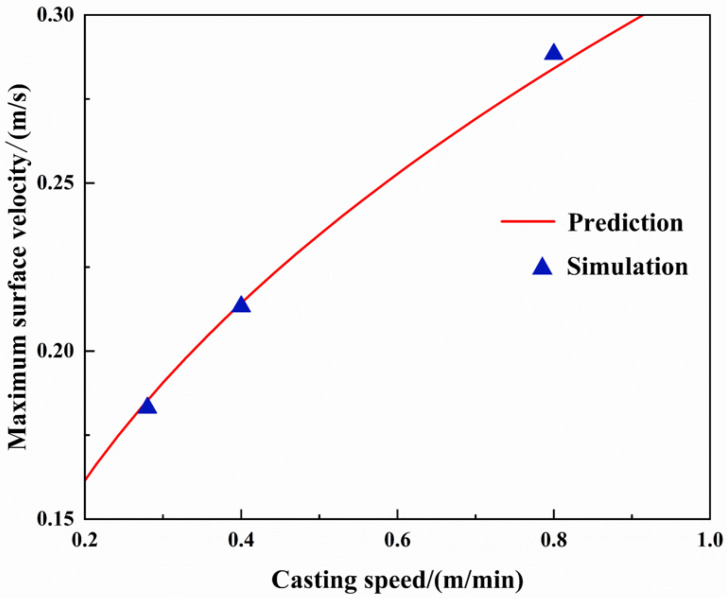
Validation of the predicted maximum velocity at the mold free surface.

As shown in Figure 17, the predicted maximum velocity at the mold free surface is validated against new numerical simulation results. Taking casting speed as an example—with other parameters fixed at a current of 150 A, frequency of 2 Hz, and SEN immersion depth of 100 mm—simulation results at casting speeds of 0.28 m/min, 0.40 m/min, and 0.80 m/min are compared with the corresponding predictions. The relative errors are 1.09%, 0.33%, and 1.47%, respectively, indicating good agreement and accuracy.

**Figure 18:**
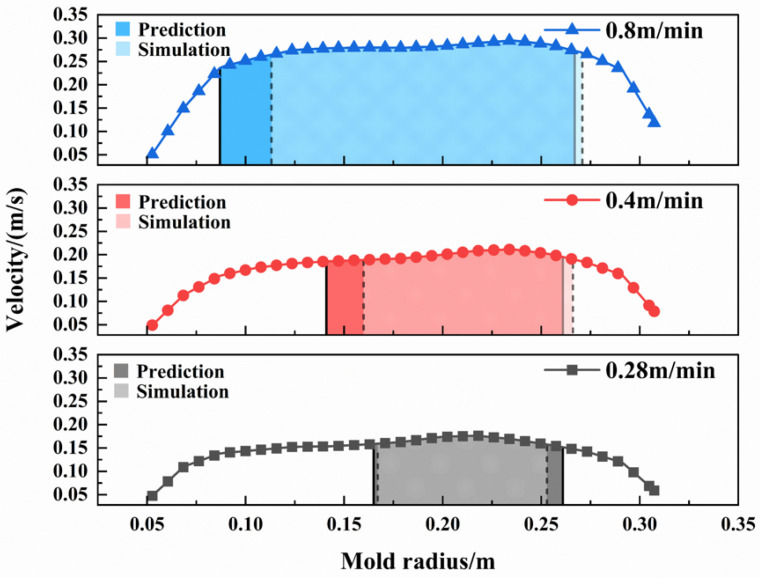
The predicted and simulated results of the high-velocity range at the mold free surface for different casting speeds.

Figure 18 presents the predicted and simulated results of the high-velocity range at the mold free surface for different casting speeds. Based on the definition of the high-velocity range established earlier, the radial positions of this range at casting speeds of 0.28 m/min, 0.40 m/min, and 0.80 m/min are determined from simulations to be (0.70 ± 0.143)*R*, (0.71 ± 0.177)*R* and (0.64 ± 0.263)*R*, respectively. In comparison, the predicted results of the regression model for the round billet mold are (0.71 ± 0.16)*R*, (0.67 ± 0.20)*R*, and (0.59 ± 0.30)*R*. The corresponding prediction accuracies are 89.58%, 84.87%, and 86.11%, which are also acceptable in engineering applications.

In this study, the CFD database was constructed using the single-parameter variation approach. Although the predictive accuracy of this method can already meet the requirements of engineering applications, it fails to effectively capture the interaction effects among parameters. Adopting more advanced multi-parameter simultaneous variation methods is expected to further improve the generalization capability of the regression model. Specifically, machine learning techniques such as Artificial Neural Networks (ANNs) or Gaussian Processes (GPs) could be introduced to better capture complex nonlinear interactions. Regarding data fitting, a power-law regression form was adopted in this study; employing more advanced techniques, such as Genetic algorithm symbolic regression, may help achieve higher fitting accuracy. Additionally, regarding turbulence modeling, the standard *k–ε* model was employed in this study to describe the turbulent flow behavior in the continuous casting mold under electromagnetic stirring (EMS). Although this model has been widely validated in previous studies and is considered applicable for simulating such flows, it inherently exhibits certain limitations in handling the strong swirling and recirculating flows induced by EMS. Employing more appropriate turbulence models, such as the Realizable *k–ε*, RNG *k–ε*, SST *k–ω*, or Reynolds Stress Model (RSM), may yield more accurate prediction results.

## 5. Conclusions

A single-phase flow model for a Ø600 mm round billet mold was established to simulate the transient flow of molten steel under various casting parameters. The velocity distribution at the mold free surface was obtained, and the internal flow field was systematically analyzed.

(1) The simulation results indicate that the maximum surface velocity in the mold is positively correlated with casting speed and EMS current, but negatively correlated with frequency, while the SEN immersion depth has a negligible effect. Specifically, at an EMS current of 300 A, the maximum surface velocity reached 0.378 m/s.
(2) By performing regression fitting on the maximum surface velocity in the mold under various parameters, a predictive regression equation for the maximum surface velocity was obtained: $v_{max}=0.00257{\;\times \;v}^{0.407}\times I^{1.009}\times f^{-0.252}\times H^{-0.0184}$.

(3) A definition for the high-velocity range was established; by fitting the median and width of this range under different parameter conditions, a predictive expression for the high-velocity range at the surface of the round billet mold was derived as $E_{W}=0.275{\;\times \;v}^{-0.175}\times I^{-0.0945}\pm 2.755{\;\times \;v}^{0.6}\times I^{-0.688}\times f^{0.23}$.

(4) Under various casting speed conditions, the predicted results for both the maximum surface velocity in the mold and the high-velocity range location were compared with the simulated results. The relative error for the former is consistently maintained within 2%, while the prediction accuracy for the latter consistently exceeds 80%. The established CFD-derived regression model enables real-time prediction of both the maximum velocity and the high-velocity range at the free surface of the Ø600 mm round billet mold.

## Figures and Tables

**Table 1:** Geometric dimensions and physical properties.

Parameters	Value
SEN immersion depth/(mm)	50, 70, 100, 130
Height of Mold/(mm)	780
Diameter of Round Billet/(mm)	600
Casting Speed/(m·min^−1^)	0.26, 0.3, 0.5, 1.0
Height of Computational Domain/(mm)	3000
Density of Steel/(kg·m^−3^)	7020
Viscosity of Steel/(kg·m^−1^·s^−1^)	0.006
Inner Diameter of Stirrer/(mm)	950
Outer Diameter of Stirrer/(mm)	1485
Height of Iron Core/(mm)	280
Current of M-EMS/(A)	100, 150, 200, 300
Frequency of M-EMS/(Hz)	1, 2, 3, 4
Iron Core relative Permeability	1000
Electrical Conductivity of Steel/(s·m^−1^)	7.14 × 10^5^

**Table 2:** Boundary conditions used in the numerical model.

Boundary	Boundary Condition Type	Value
Inlet of mold	Velocity inlet	1.04/1.20/2.00/4.00 m·s^−1^
Top surface	Specified shear	0 Pa
Outlet of mold	Pressure outlet	1 atm
Inner wall of SEN	No-slip wall	-
Outer wall of SEN	No-slip wall	-
Wall of mold	No-slip wall	-

**Table 3:** Simulation error statistics in max mold surface velocity for different mesh sizes.

Mesh	Number of Hexahedral Cells	Max Velocity at the Mold Surface (m/s)	Variation of the Max Velocity (m/s)	Relative Error (%)
M_1_	287,616	0.0013	-	-
M_2_	390,720	0.00142	0.00012	9.23
M_3_	503,550	0.00155	0.00013	9.15
M_4_	756,204	0.00162	0.00007	4.52

**Table 4:** High-velocity ranges near the free surface in the mold under different casting parameters.

Parameter	Value	High-Velocity Range/m	High-Velocity Range Width/m
Casting speed /(m/min)	0.26	(0.68 ± 0.12)*R*	0.23*R*
	0.3	(0.70 ± 0.13)*R*	0.26*R*
	0.5	(0.65 ± 0.24)*R*	0.48*R*
	1.0	(0.57 ± 0.31)*R*	0.62*R*
EMS current /A	100	(0.75 ± 0.16)*R*	0.32*R*
	150	(0.68 ± 0.12)*R*	0.23*R*
	200	(0.70 ± 0.09)*R*	0.18*R*
	300	(0.68 ± 0.08)*R*	0.16*R*
EMS frequency /Hz	1	(0.68 ± 0.11)*R*	0.22*R*
	2	(0.68 ± 0.12)*R*	0.23*R*
	3	(0.69 ± 0.12)*R*	0.24*R*
	4	(0.67 ± 0.15)*R*	0.29*R*
SEN immersion depth/mm	50	(0.63 ± 0.13)*R*	0.26*R*
	70	(0.69 ± 0.13)*R*	0.25*R*
	100	(0.68 ± 0.12)*R*	0.23*R*

## Data Availability

The original contributions presented in this study are included in the article. Further inquiries can be directed to the corresponding authors.

## Abbreviations

The following abbreviations are used in this manuscript:

EMS	Electromagnetic Stirring
SEN	Submerged Entry Nozzle
CFD	Computational Fluid Dynamics
